# Supplementary material for: Evaluating the effectiveness of preservice midwifery curricula in Ethiopia: A comparison of neonatal resuscitation and infection prevention practice of midwifery graduates trained in competency-based versus conventional curricula
Source: PLoS One. 2026 Feb 10;21(2):e0338395. doi: 10.1371/journal.pone.0338395 (PMC12890084; doi:10.1371/journal.pone.0338395)
Supplement: S1 Fig — (DOCX) [file pone.0338395.s002.docx]

**Supplement 1: Figure 1 Study procedure and workflow for the labor and delivery observations** (adopted by the research team).

**Supervisor**

**Arrives at health facility where service provider works**

**Service provider enrolled in the study**

**Data collectors**

**Completion of screening form**

**Service provider consent**

**Screening & pre-eligibility checks**

**Arrives at health facility where service provider works**

**Observation starts**

**Women arrive at health facility & admitted for childbirth**

**Women in labor**

**Women give birth**

Women

**Women**

**Women screening,**

**Obtain women consent**

**Women enrolled in the study**

**Service provider**

**Labor & Delivery observation form completed,**

**End of data collection: 1 hour postpartum**

**Central data management team**

**Consistency data check**

**Data cleaning**

**Final database**

**Data auditing & monitoring**

**Daily screening and data submission**

**Data consistency report**

**Regular email and phone communication**

Data consistency

Regual communication with data collectors
