# Supplementary material for: Evaluating the effectiveness of preservice midwifery curricula in Ethiopia: A comparison of neonatal resuscitation and infection prevention practice of midwifery graduates trained in competency-based versus conventional curricula
Source: PLoS One. 2026 Feb 10;21(2):e0338395. doi: 10.1371/journal.pone.0338395 (PMC12890084; doi:10.1371/journal.pone.0338395)
Supplement: S1 Table — (DOCX) [file pone.0338395.s003.docx]

**Supplement 2: Background of clients observed and characteristics of health facilities**

| **Clients (*N* = 68)** | **Category** | **Count**  **(total = 68)** | **Percentage** |
| --- | --- | --- | --- |
| Residence | Urban | 44 | 64.7 |
|  | Rural | 24 | 35.3 |
| Maternal age (years) | 20-24 | 4 | 5.9 |
|  | 25-29 | 29 | 42.6 |
|  | 30-34 | 30 | 44.1 |
|  | 35 and above | 5 | 7.4 |
|  | All | 29.3 | 3.0 |
| Marital status | Not married | 6 | 8.8 |
|  | Married | 62 | 91.2 |
| Gravida | 1 | 6 | 8.8 |
|  | 2-4 | 49 | 72.1 |
|  | 5 and more | 13 | 19.1 |
| Parity | ≤4 | 61 | 89.7 |
|  | ≥5 | 7 | 11.3 |
| **Health Facilities (*N* = 29)** | | | |
| Type of facility | Referral Hospital | 4 | 13.8 |
|  | General Hospital | 2 | 6.9 |
|  | Primary Hospital | 4 | 13.8 |
|  | Health Center | 19 | 65.6 |
| Place of facility | Urban | 20 | 68.9 |
|  | Rural | 9 | 31.0 |
| Volume of deliveries | < 5/day | 19 | 65.5 |
|  | ≥ 5/day | 10 | 34.5 |
