# Supplementary material for: Evaluating the effectiveness of preservice midwifery curricula in Ethiopia: A comparison of neonatal resuscitation and infection prevention practice of midwifery graduates trained in competency-based versus conventional curricula
Source: PLoS One. 2026 Feb 10;21(2):e0338395. doi: 10.1371/journal.pone.0338395 (PMC12890084; doi:10.1371/journal.pone.0338395)
Supplement: Attachment — Submitted filename: Response to Reviewers III.docx [file pone.0338395.s005.docx]

**To:** The Editorial Office

**PLOS ONE**

**Subject:** Submission of Revised Manuscript “***Evaluating the effectiveness of preservice midwifery curricula in Ethiopia: A comparison of neonatal resuscitation and infection prevention practice of midwifery graduates trained in competency-based versus conventional curricula (PONE-D-25-43421*)**”

Dear Editors,

On behalf of all authors, I am pleased to submit the revised version of our manuscript entitled *“Evaluating the effectiveness of preservice midwifery curricula in Ethiopia: A comparison of neonatal resuscitation and infection prevention practice of midwifery graduates trained in competency-based versus conventional curricula”* for consideration in **PLOS ONE**.

We thank the reviewers and editor for their thoughtful feedback and constructive suggestions. In this revision, we have addressed all comments in detail, including:

- Clarifying methodology and sampling rationale, including inclusion/exclusion criteria and representativeness of third-generation Ethiopian midwifery graduates.
- Expanding the ethical considerations section with clear information on oral and written informed consent, confidentiality, and voluntary participation.
- Providing transparent statistical reporting, including effect sizes, confidence intervals, and multiple comparison corrections, along with visualizations via Gardner–Altman plots.
- Strengthening discussion and conclusion sections to explicitly link findings to actionable policy recommendations and midwifery workforce development.
- Revising language throughout for clarity, conciseness, and consistent terminology.

We believe that these revisions substantially improve the clarity, rigor, and impact of the manuscript. We are confident that this study provides important evidence on the effectiveness of competency-based midwifery curricula in Ethiopia and contributes to the global literature on the development of the maternal and newborn health workforce.

This manuscript is original, has not been published elsewhere, and is not under consideration by any other journal. All authors have reviewed and approved the final version.

**Updated Competing Interests Statement:**

Awoke Giletew Wondie and Tegbar Yigzaw Sendekie were involved in the design and implementation of the competency-based curriculum at Debre Tabor University. To minimize bias, these authors did not participate in the data collection, observation, or scoring processes. Data collection was conducted by trained independent midwife observers who were blinded to the graduates’ curriculum background. Author TYS is employed by Jhpiego's Health Workforce Improvement Project. This employment provided salary support but did not influence the study design, data collection and analysis, decision to publish, or preparation of the manuscript. There are no patents, products in development, marketed products, or consultancies related to this research. This does not alter our adherence to PLOS ONE policies on sharing data and materials.

**Updated Funding Statement**

This study was supported by Jhpiego's Health Workforce Improvement Project. Author TYS is employed by Jhpiego's Health Workforce Improvement Project. The funder provided support in the form of salaries for TYS but did not have any additional role in study design, data collection and analysis, decision to publish, or preparation of the manuscript. The specific role of this author is articulated in the ‘Author Contributions’ section.

We have also included a detailed, ***31-page*** point-by-point response to all editorial and reviewer comments, which we hope will facilitate your evaluation of the revised manuscript.

We greatly appreciate your time and consideration and look forward to your favorable response.

Sincerely,
on behalf of all co-authors,

**Awoke Giletew Wondie, MHPE, MPH**

PhD Candidate in Medical Research – International Health

Ludwig-Maximilians-Universität München

**Journal Requirements 1:**

When submitting your revision, we need you to address these additional requirements.

1. Please ensure that your manuscript meets PLOS ONE's style requirements, including those for file naming. The PLOS ONE style templates can be found at:

<https://journals.plos.org/plosone/s/file?id=wjVg/PLOSOne_formatting_sample_main_body.pdf> and

<https://journals.plos.org/plosone/s/file?id=ba62/PLOSOne_formatting_sample_title_authors_affiliations.pdf>

**Response:** We have carefully revised the manuscript to comply with **PLOS ONE formatting guidelines**, including the main text, title page, headings, figure and table formats, and reference style. File names have been updated in accordance with the journal's instructions.

2. Please include a complete copy of PLOS’ questionnaire on inclusivity in global research in your revised manuscript. Our policy for research in this area aims to improve transparency in the reporting of research performed outside of researchers’ own country or community. The policy applies to researchers who have travelled to a different country to conduct research, research with Indigenous populations or their lands, and research on cultural artefacts. The questionnaire can also be requested at the journal’s discretion for any other submissions, even if these conditions are not met. Please find more information on the policy and a link to download a blank copy of the questionnaire here: https://journals.plos.org/plosone/s/best-practices-in-research-reporting.

Please upload a completed version of your questionnaire as Supporting Information when you resubmit your manuscript.

**Response:** We appreciate the reviewer’s request regarding the Inclusivity in Global Research questionnaire. The corresponding author (Awoke Giletew) is affiliated with Debre Tabor University (DTU), which is located in Ethiopia and is also the site of this research. As such, the research was conducted within the authors’ own institution and country, and not as external researchers.

- In line with PLOS ONE’s policy, we have nevertheless completed the Inclusivity in Global Research questionnaire to ensure transparency.
- The completed questionnaire has been uploaded as Supporting Information (***S1 Questionnaire***) in the revised submission.
- We have also updated the Supporting Information section of the manuscript to include a reference to this questionnaire.

**Revisions includes:**

**Supporting information**

- S1 Questionnaire. Completed Inclusivity in Global Research Questionnaire.

**Revised affiliation:**

Awoke Giletew Wondie^1,2^*, Matthias Siebeck^1,3^, Tegbar Yigzaw Sendekie^4^, Martin Fischer ^3^, Markus Berndt ^3^

^1^ CIH LMU, Center for International Health, LMU University Hospital, LMU Munich, Germany

^2^ Department of Reproductive and Child Health, College of Health Sciences, Debre Tabor University, Debre Tabor, Ethiopia

^3^ Institute of Medical Education, LMU University Hospital, LMU Munich, Germany

^4^ Jhpiego (an affiliate of John Hopkins University), Addis Abeba, Ethiopia

3. In the ethics statement in the Methods, you have specified that verbal consent was obtained. Please provide additional details regarding how this consent was documented and witnessed, and state whether this was approved by the IRB.

**Response:** We appreciate this important comment. We have now clarified the process by which verbal consent was obtained, documented, and approved by the Institutional Review Board (IRB). Specifically:

- Verbal informed consent was obtained because some participants had limited literacy.
- The consent process was documented by the data collectors, who signed a form confirming that the participant had received and understood the study information and had voluntarily agreed to participate.
- Each consent process was witnessed by a colleague from the health facility where the participant was working.
- This procedure for verbal consent, including documentation and witnessing, was reviewed and formally approved by the IRB.

We have revised the **Ethics statement** in the Methods section to reflect these details.

**Revised Ethics Statement (Methods section):**

*“The study protocol was approved by the Ethical Review Committee of Debre Tabor University, Ethiopia (Date: 13.07.2023 / No: RP/251/23), and the Ethics Commission of the Medical Faculty of Ludwig-Maximilians-Universität, Munich, Germany (Date: 18.10.2023 / Project Nr.: 23-0757). At each health facility, the supervisor presented an official letter from DTU to the facility director requesting cooperation. Written consent was obtained from facility directors, while oral consent was obtained from midwives and laboring women. Written consent was not collected from midwives due to their engagement in clinical care, although the potential benefits and burdens had been discussed beforehand. For laboring women, written consent was waived owing to literacy challenges and to minimize the burden during labor.*

*Data collectors were trained to approach participants respectfully, explain the study purpose, and obtain informed consent, including discussion of potential risks and benefits. Providers were assured that participation or refusal would not affect their employment, and women were assured that nonparticipation would not influence the care they received. Participant and facility confidentiality was strictly maintained: no names of facilities, service providers, or clients were recorded, and results were reported anonymously to prevent identification.*

*All informed consent procedures were reviewed and approved by the respective ethics committees.”*

4. Thank you for stating the following in the Competing Interests section:

“Conflict of interest

Awoke Giletew Wondie and Tegbar Yigzaw Sendekie designed and implemented the competency-based curriculum at Debre Tabor University. However, these authors had no part in the collection or scoring of data.  The assessment was conducted by trained independent midwife observers blinded to the graduates' curriculum background being evaluated.”

**Response:** We thank the reviewer for acknowledging our competing interest statement. To improve transparency, we have slightly revised the section to align more closely with *PLOS ONE*’s **Competing Interests policy**. In the revision, we explicitly declare the authors’ involvement in curriculum design and clarify that this does not alter adherence to PLOS ONE’s policies on data sharing and research integrity as follows:

*“****Updated Competing Interests Statement:***

*Awoke Giletew Wondie and Tegbar Yigzaw Sendekie were involved in the design and implementation of the competency-based curriculum at Debre Tabor University. To minimize bias, these authors did not participate in the data collection, observation, or scoring processes. Data collection was conducted by trained independent midwife observers who were blinded to the graduates’ curriculum background. Author TYS is employed by Jhpiego's Health Workforce Improvement Project. This employment provided salary support but did not influence the study design, data collection and analysis, decision to publish, or preparation of the manuscript. There are no patents, products in development, marketed products, or consultancies related to this research. This does not alter our adherence to PLOS ONE policies on sharing data and materials.”*

**Journal Requirements 2:**

1. We note that one or more of the authors are employed by a commercial company: name of commercial company.

Please provide an amended Funding Statement declaring this commercial affiliation, as well as a statement regarding the Role of Funders in your study. If the funding organization did not play a role in the study design, data collection and analysis, decision to publish, or preparation of the manuscript and only provided financial support in the form of authors' salaries and/or research materials, please review your statements relating to the author contributions, and ensure you have specifically and accurately indicated the role(s) that these authors had in your study. You can update author roles in the Author Contributions section of the online submission form.

Please also include the following statement within your amended Funding Statement.

“The funder provided support in the form of salaries for authors [insert relevant initials], but did not have any additional role in the study design, data collection and analysis, decision to publish, or preparation of the manuscript. The specific roles of these authors are articulated in the ‘author contributions’ section.”

If your commercial affiliation did play a role in your study, please state and explain this role within your updated Funding Statement.

2. Please also provide an updated Competing Interests Statement declaring this commercial affiliation along with any other relevant declarations relating to employment, consultancy, patents, products in development, or marketed products, etc.

Within your Competing Interests Statement, please confirm that this commercial affiliation does not alter your adherence to all PLOS ONE policies on sharing data and materials by including the following statement: "This does not alter our adherence to PLOS ONE policies on sharing data and materials.” (as detailed online in our guide for authors http://journals.plos.org/plosone/s/competing-interests) . If this adherence statement is not accurate and there are restrictions on sharing of data and/or materials, please state these. Please note that we cannot proceed with consideration of your article until this information has been declared.

Please include both an updated Funding Statement and Competing Interests Statement in your cover letter. We will change the online submission form on your behalf.

**Response:**
We thank the editors for these comments. We confirm and revised in the cover letter as follow:

***“Updated Competing Interests Statement:***

*Awoke Giletew Wondie and Tegbar Yigzaw Sendekie were involved in the design and implementation of the competency-based curriculum at Debre Tabor University. To minimize bias, these authors did not participate in the data collection, observation, or scoring processes. Data collection was conducted by trained independent midwife observers who were blinded to the graduates’ curriculum background. Author TYS is employed by Jhpiego's Health Workforce Improvement Project. This employment provided salary support but did not influence the study design, data collection and analysis, decision to publish, or preparation of the manuscript. There are no patents, products in development, marketed products, or consultancies related to this research. This does not alter our adherence to PLOS ONE policies on sharing data and materials.*

***Updated Funding Statement***

*This study was supported by Jhpiego's Health Workforce Improvement Project. Author TYS is employed by Jhpiego's Health Workforce Improvement Project. The funder provided support in the form of salaries for TYS but did not have any additional role in study design, data collection and analysis, decision to publish, or preparation of the manuscript. The specific role of this author is articulated in the ‘Author Contributions’ section.”*

3. If the reviewer comments include a recommendation to cite specific previously published works, please review and evaluate these publications to determine whether they are relevant and should be cited. There is no requirement to cite these works unless the editor has indicated otherwise.

**Response:** We thank the reviewer for suggesting additional references. We have carefully reviewed the recommended publications and evaluated their relevance to our study.

Please review your reference list to ensure that it is complete and correct. If you have cited papers that have been retracted, please include the rationale for doing so in the manuscript text, or remove these references and replace them with relevant current references. Any changes to the reference list should be mentioned in the rebuttal letter that accompanies your revised manuscript. If you need to cite a retracted article, indicate the article’s retracted status in the References list and also include a citation and full reference for the retraction notice.

Response: We have thoroughly reviewed all references to ensure their completeness and accuracy. All references now contain full bibliographic details, including DOIs and PMCID numbers where available. We confirmed that no articles cited in the study have been retracted. Minor corrections to formatting were made. All changes have been highlighted in the revised manuscript.

**Response to Reviewer Comments**

**Reviewer 1**

Reviewer #1: This manuscript presents a well-designed study comparing midwifery curricula in Ethiopia, addressing a critical gap in medical education literature for low- and middle-income countries. The findings are compelling and have significant implications for public health and curriculum design. The manuscript is well-written and methodologically sound, providing strong evidence that competency-based curricula improve neonatal care skills. While highlighting persistent skill gaps in both graduate groups, the study is a valuable contribution.

**Major Strengths**

**High-Impact Research**: The study addresses the crucial issue of how-to best train healthcare providers to improve maternal and neonatal outcomes, providing concrete evidence to support a global shift toward competency-based medical education.

**Robust Methodology**: The study's rigor is enhanced by its comparative design, formal power analysis, use of validated tools, blinding of data collectors, and employment of two independent observers.

**Strong Data Analysis**: Statistical analysis is appropriate and strengthened by the inclusion of Gardner-Altman plots for modern visualization of the effect size.

**Response:**

We sincerely thank the reviewer for the encouraging comments and positive assessment of our work. We greatly appreciate the recognition of the study’s relevance, methodological rigor, and contribution to the field of medical education and maternal-newborn care.

To emphasize the study’s contribution and implications, we have added clarifying sentences in the **Introduction** and **Discussion** sections, highlighting the global relevance of the findings.

**Areas for Improvement**

1. Clarity & Focus: The thesis should be reframed as a debatable argument about the curriculum's effectiveness.

**Response**: We appreciate the reviewer's constructive suggestion. We agree that framing the study around a clear, debatable thesis strengthens the manuscript’s focus. Accordingly, we have revised the **Introduction** to explicitly present the research question as a testable argument: whether competency-based curricula improve midwifery graduates’ performance in neonatal resuscitation and infection prevention compared to conventional curricula.

1. **Introduction (Beginning of paragraph 3):**

**Original:**

*“This study investigates whether such curriculum reform enhances the provision of neonatal resuscitation and infection prevention services by comparing graduates from competency-based and conventional programs.”*

**Revised:**

*“This study tests the hypothesis that graduates of competency-based midwifery curricula demonstrate superior performance in neonatal resuscitation and infection prevention compared to those trained under conventional curricula, thereby framing a clear, debatable argument about the curriculum’s effectiveness in preparing clinically competent midwives.”*

2. **Abstract – background section:**

**Original:**

*“This study compares the performance of competency-based curriculum graduates (CBCGs) and conventional curriculum graduates (CCGs) in these essential practices.”*

**Revised:**

*“This study examines whether competency-based midwifery education produces graduates with significantly better performance in neonatal resuscitation and infection prevention compared to conventional education, thereby framing a testable argument about the curriculum’s effectiveness.”*

3. **Conclusion – Reframing around the debatable thesis**

**Original:**

*“This study provides evidence that competency-based midwifery education significantly improves graduates' performance in neonatal resuscitation and infection prevention compared to conventional training. The observed differences are both statistically and practically meaningful, reflecting enhanced clinical readiness among graduates of a competency-based curriculum. Nonetheless, persistent deficiencies in essential tasks across both groups highlight the need for continued curriculum improvement and stronger clinical training systems.”*

**Revised:**

*“This study demonstrates that competency-based midwifery education can improve graduates’ performance in neonatal resuscitation and infection prevention relative to conventional curricula. However, the persistence of skill gaps in both groups highlights that curriculum effectiveness is not absolute and may depend on the quality of implementation, mentorship, and reinforcement of practice. These findings frame a testable and debatable argument: while competency-based curricula have the potential to enhance clinical competence, ongoing evaluation, targeted improvements, and context-specific adaptations are essential to fully realize their effectiveness in improving maternal and neonatal outcomes.”*

2. Structure & Detail: Improve transitions by more explicitly linking the introduction's critique of theory-based education to the competency-based curriculum. The Methods section needs more detail on evaluation criteria and observer training to improve replicability. The section on infection prevention should be expanded to discuss key practices like hand hygiene and sterile technique.

**Response**: We appreciate the reviewer's constructive suggestions. We agree that improving transitions, adding methodological detail, and expanding discussion on infection prevention will enhance clarity, replicability, and relevance. Accordingly, we have made the following revisions:

**1. Introduction – Linking theory-based education to competency-based curriculum**

**Original:**

*“In Ethiopia, the emphasis on theory of university midwifery education has limited graduates' practical competencies. In response, Debre Tabor University (DTU) adopted a competency-based integrated curriculum in 2013, incorporating problem-based learning, early clinical exposure, simulation-based education, and community-based education (5).”*

**Revised:**

*“In Ethiopia, traditional theory-heavy midwifery education has resulted in graduates with limited clinical competence, particularly in essential life-saving procedures. To address these gaps, Debre Tabor University (DTU) implemented a competency-based integrated curriculum in 2013, explicitly designed to translate theoretical knowledge into practical skills through problem-based learning, early clinical exposure, simulation-based training, and community-based education. This curriculum represents a targeted response to the deficiencies of conventional programs, directly linking educational reform to improved clinical performance (5).”*

**2. Methods – Adding detail on evaluation criteria and observer training**

**Revised:**

*“Each task was scored as ‘performed correctly’ or ‘not performed/correctly’ to calculate a percentage performance score. Observers underwent a five-day training program that covered study protocols, detailed task scoring criteria, interrater calibration, and role-play exercises to ensure consistency. Observers were blinded to participants’ curriculum group to minimize bias. Average scores from two independent observers were used for analysis, enhancing the reliability and replicability of the evaluation.”*

**3. Methods & Results – Expanding infection prevention discussion**

**Method section - Revised:**

*“It was assessed in detail, focusing on critical components, including proper hand hygiene before and after procedures, the use of sterile gloves, the appropriate use of personal protective equipment (PPE), instrument decontamination, the safe disposal of sharps and waste, and the proper removal and disinfection of aprons.”*

**Revised (Results, Infection Prevention):**

*“Overall, midwives implemented 71.7% (95% CI: 68.1–75.3) of infection prevention tasks. Universal glove use and proper waste disposal were consistently observed, highlighting that while basic infection prevention measures are routinely applied, critical components of sterile technique revealed gaps in key practices, including hand hygiene (48.6%), PPE use (83.8%), and apron decontamination (11.8%) (****Table 3****).*

*Comparing group performance, CBCGs scored higher (76.9%) than those trained under the conventional curriculum (67%). The key differences were observed in hand hygiene (62.5% vs 36.1%), PPE use (90.6% vs. 77.8%), and apron decontamination (18.7% vs. 5.6) (****Table 3****).”*

3. Discussion & Context: The discussion should further analyze why skill deficiencies persist in both groups. It should also acknowledge the limitations of using simulation for assessment and consider how post-graduation work environments might influence graduate performance.

**Response:** We appreciate the reviewer's valuable feedback. We agree that analysing persistent skill gaps, acknowledging simulation limitations, and considering workplace factors will strengthen the discussion and contextualize our findings. We have revised the Discussion section accordingly.

**1. Persistent skill deficiencies**

**The following revision was incorporated in the discussion:**

*“Despite overall improvements among CBCGs, persistent deficiencies were observed in both groups, particularly in critical steps such as checking breathing/pulse during neonatal resuscitation and proper removal and disinfection of aprons. These gaps may result from limited opportunities for repeated hands-on practice during training, retention challenges over time, and variability in clinical supervision. Additionally, graduates' performance may also be influenced by post-graduation work environments, such as staffing levels, equipment availability, and supervisory support, which may contribute to observed skill deficiencies, suggesting that curriculum effectiveness is influenced by the workplace context.”*

**2. Limitations of using simulation for assessment**

**The following limitation was incorporated in the discussion:**

*“While simulation-based assessment provides a controlled environment to evaluate neonatal resuscitation skills, it may not fully capture performance under real-life clinical pressure. Graduates may demonstrate higher performance in simulation than in actual labor settings, or conversely, may underperform due to unfamiliarity with the simulated environment. Thus, results should be interpreted with caution, recognizing that simulation outcomes may overestimate or underestimate true clinical competence.”*

**3. Influence of post-graduation work environments**

**The following revision was incorporated in the discussion:**

*“Additionally, graduates' performance may also be influenced by post-graduation work environments, such as staffing levels, equipment availability, and supervisory support, which may contribute to observed skill deficiencies, suggesting that curriculum effectiveness is influenced by the workplace context.”*

**Minor Points & Corrections**

4. Fix the mismatch between figure captions in the text and the actual figures.

**Response:** We thank the reviewer for bringing this to our attention. We carefully reviewed all figure captions and the corresponding figures and have corrected mismatches to ensure consistency.

**Original:**

*“Fig 1. A Gardner-Altman plot estimating the difference in infection prevention practice.”*

*And*

*“**Fig 2. A Gardner-Altman Plot estimating the difference in neonatal resuscitation”*

**Corrected caption:**

*“Fig1. A Gardner-Altman plot estimating the difference in neonatal resuscitation performance between competency-based and conventional curriculum graduates.”*

*And*

*“Fig 2. A Gardner-Altman plot estimating the difference in infection prevention practice between competency-based and conventional curriculum graduates.”*

All figure references in the **Results and Discussion** sections have been cross-checked to ensure that each figure number corresponds accurately to its content and caption.

5. Consolidate the redundant paragraphs in the "Suggestions" section.

**Response:** We appreciate the reviewer's helpful observation. We have consolidated the redundant paragraphs in the ***Suggestions*** section to enhance clarity and avoid repetition while maintaining all key recommendations for improving midwifery education. It is indicated as follows:

*“To address the remaining gaps in clinical performance, midwifery education programs should strengthen the implementation of competency-based approaches by incorporating more frequent simulation exercises, structured clinical mentorship, and targeted feedback mechanisms. Ensuring a strong alignment between theoretical content and hands-on practice is essential. Additionally, periodic refresher training should be considered to maintain and enhance clinical skills over time.”*

**Reviewer 2**

Reviewer #2: This manuscript addresses an important and under-researched area: the assessment of midwifery competencies in Ethiopia, benchmarked against the International Confederation of Midwives (ICM) Essential Competencies. Given Ethiopia’s continuing maternal and neonatal health burden and the global push to strengthen human resources for health, this study is timely and policy relevant. The use of an international competency framework is a major strength, and the findings could inform reforms in pre-service education, continuing professional development, and regulation of the midwifery profession.

However, the manuscript requires significant revision before it can be considered for publication. The most critical issues relate to methodological clarity, statistical transparency, and the explicit linkage between findings and policy/practice. Without addressing these areas, the paper’s impact and credibility are limited.

1. Technical soundness: The manuscript is based on empirical data collection and analysis, and in general, the conclusions are aligned with the findings. The description of midwifery competencies and the assessment framework corresponds with international standards. However, the linkage between data and conclusions needs to be strengthened by clarifying how competency gaps were identified (e.g., objective testing, self-assessment, observation, or supervisor reports), providing justification for sample size and its representativeness across Ethiopia’s diverse regions, and indicating whether clinical skills testing (e.g., OSCE) was used or if the assessment was limited to theoretical knowledge.

**Response:** We thank the reviewer for recognizing the study’s empirical rigor and alignment with international competency standards. We agree that clarifying the assessment methods, justifying the sample size, and outlining the testing approach will strengthen methodological transparency and the linkage between data and conclusions. Accordingly, the following clarifications and revisions have been made in the **Methods** section.

**A. Clarification on how competency gaps were identified:**

*”The primary outcomes were the performance of neonatal resuscitation and infection prevention practice at birth (****S1. Dataset****). Data was collected through* ***direct structured observation*** *of labor and deliveries to assess infection prevention practices and evaluate midwives' newborn resuscitation skills in a simulated environment using the NeoNatalie model (7).*

*Adherence to infection prevention practices was measured using a standardized tool from the Maternal, Newborn, and Child Health Integrated Program Quality of Care Assessment for Labor, Delivery, and Newborn Care (8). It was assessed in detail, focusing on critical components, including proper hand hygiene before and after procedures, the use of sterile gloves, the appropriate use of personal protective equipment (PPE), instrument decontamination, the safe disposal of sharps and waste, and the proper removal and disinfection of aprons. Neonatal resuscitation performance was evaluated through a validated, competency-based simulation assessment of resuscitation skills (9).*

*Two trained, independent observers assessed participants’ performance during simulated neonatal resuscitation and labor and delivery sessions. Each task was scored as ‘performed correctly’ or ‘not performed/correctly’ to calculate a percentage performance score. Observers underwent a five-day training program that covered study protocols, detailed task scoring criteria, interrater calibration, and role-play exercises to ensure consistency and reliability. Observers were blinded to participants’ curriculum group to minimize bias. Average scores from two independent observers were used for analysis, enhancing the reliability and replicability of the evaluation.”*

**B. Justification for sample size and representativeness**

**Revised sample size and selection of study participants’ section:**

*“The number of participants was calculated based on a power analysis to determine a moderate effect size (d = 0.5) with 80% power and α = .05, ensuring sufficient power to detect a medium-to-large effect size between groups, resulting in 32 participants per group. Of 37 recent DTU graduates, 32 met the inclusion criteria; five were excluded due to unemployment, career change, or working outside the study area. Three of the nine third-generation universities (excluding DTU) were excluded because two had already started implementing similar curricula, and one lacked sufficient data. To account for anticipated non-response, a 10% margin of error was added, resulting in a final sample of 36 randomly selected participants from the comparison group's registrar lists. Participants were employed across all regions of Ethiopia, and the inclusion of both public hospitals and health centers serving urban and rural populations enhances contextual representativeness.”*

**C. Specification of clinical skills testing (OSCE vs. theoretical assessment)**

**Response**: No written or theoretical knowledge tests were administered, as the study focused on direct performance-based assessment of practical competencies. Therefore, midwives' performance was measured by direct structured observation of labor and deliveries (infection prevention), and direct structured observation of newborn resuscitation skills in a simulated environment, but not OSCE, which allows for administering multiple stations.

It is indicated in the revised manuscript clearly as follows:

*“Data was collected through* ***direct structured observation*** *of labor and deliveries to assess infection prevention practices and evaluate midwives' newborn resuscitation skills in a simulated environment using the NeoNatalie model (7).”*

2. Statistical analysis: The manuscript uses descriptive and inferential statistics, but the methodology is not fully transparent. For examples: Page 7, lines 14–30; Page 9, lines 1–20 – statistical outputs are presented without clear explanation of assumptions, significance tests, or confidence intervals.

Suggestions:
- Specify the statistical methods, including rationale for choice of tests, assumptions checked, and the software used.

- Report measures of reliability (e.g., Cronbach’s alpha for internal consistency).
- Without these details, it is difficult to assess the rigor of the analysis.

**Response:** We appreciate the reviewer's thoughtful comment. We agree that transparency in describing statistical procedures and reliability testing is essential.

While the original manuscript already included key statistical procedures, such as use of **IBM SPSS v27**, **independent t-tests**, **Cohen’s d with Gardner–Altman plots**, and **multiple comparison corrections** (Bonferroni, Holm–Bonferroni, and FDR), we have now clarified the following to enhance clarity and rigor:

A. **Added explicit details** about assumption checks for normality and homogeneity of variance.

**Revised section (2.5 Data analysis):**

*“Data were analysed using IBM SPSS Statistics version 27. Performance was measured as the percentage mean score for each outcome (neonatal resuscitation and infection prevention). Independent t-tests were used to compare graduates from competency-based and conventional curricula, with effect sizes (Cohen’s d) visualized by Gardner–Altman plots. The assumptions of normality and homogeneity of variance were verified using the Shapiro–Wilk and Levene’s tests, respectively. These outcomes were part of four primary outcomes assessed in a broader study. Multiple comparison corrections were applied using Bonferroni, Holm–Bonferroni, and false discovery rate methods to account for related outcomes. Both results remained statistically significant under all adjustments.”*

B. Introduced a dedicated subsection detailing **interrater reliability and internal consistency** of the instruments.

**New subsection, Interrater Reliability and Internal Consistency, added after Data Analysis subsection:**

*“Although previously validated in a similar setting, the tool’s internal consistency was re-evaluated using data from this study. Interrater reliability of the total performance scores was assessed using a two-way mixed effects intraclass correlation coefficient (ICC) with a consistency definition. The ICC for average measures of infection prevention and neonatal resuscitation were 0.87 (95% CI: 0.79–0.92, p < 0.001) and 0.84 (95% CI: 0.74–0.90, p < 0.001), respectively, suggesting excellent agreement between the two raters. Additionally, Cronbach’s alpha for infection prevention and neonatal resuscitation items was 0.66 and 0.64, respectively, indicating acceptable internal consistency for the purpose of this study.”*

C. **Results – confirmed reporting (Section 3.4)**

The Results section (3.4, Table 4, and Figures 1–2) already presents **mean scores, SDs, t-values, df, p-values, and 95% confidence intervals** for both outcomes. We confirmed that all comparisons met the assumptions for parametric testing, and the results included 95% confidence intervals to indicate the precision of estimates.

3. Language/Clarity: The manuscript is written in standard academic English, but the readability could be improved with editing. For examples:

Page 4, line 12: “which was highly important to consider” → revise to “which was essential to consider.”

Page 8, line 22: “respondents were asked competencies” → revise to “respondents were asked about competencies.”

A professional language revision is recommended to improve flow, grammar, and clarity.

**Response:**

We thank the reviewer for highlighting the importance of clear and professional language. Upon careful review, we confirmed that the specific phrases cited by the reviewer were ***not present in the submitted manuscript***. Nonetheless, we conducted a **comprehensive professional language edit** throughout the manuscript to enhance readability, flow, and academic clarity. Specific actions include:

1. **Improved sentence structure and word choice** across sections (Introduction, Methods, Results, and Discussion) to ensure concise, clear, and formal academic English.
2. **Corrected minor grammatical inconsistencies**, punctuation, and tense usage where necessary.
3. **Ensured logical flow between paragraphs and sections**, particularly linking background, methods, and findings to the study objectives.
4. **Maintained accuracy and technical precision** of all reported results, statistical outputs, and interpretations.

**Major Comments**

1. Methodology Clarity (Page 5, lines 1–25): The manuscript does not specify the exact data collection tools. Were competencies assessed via self-report questionnaires, supervisor/peer evaluations, or OSCEs. The validity and reliability of the tool must be explicitly stated. Without this, the robustness of the findings is unclear.

**Response:**

While the original manuscript already included data collection tools and procedures, we appreciate the reviewer’s comment (*In the revised manuscript, we incorporated the validity and reliability of the tool*) and provide the following clarifications, which were presented in the original manuscript:

**Data collection methods:**

- **Infection prevention practice** was assessed through **direct observation of labor and deliveries** using a standardized tool adapted from the *Maternal, Newborn, and Child Health Integrated Program Quality of Care Assessment for Labor, Delivery, and Newborn Care* (Tripathi et al., 2015).
- **Neonatal resuscitation performance** was assessed through **direct observation of midwives performing resuscitation in a simulated environment** using the NeoNatalie newborn simulator (Laerdal Global Health, 2024).
- No self-report questionnaires or peer/supervisor evaluations were used; all competencies were measured via structured, direct observation.

**Observer training and standardization:**

- Data collectors were **master’s-level clinical midwives** with over one year of maternal/newborn care experience and no affiliation with study sites.
- A **five-day training** included presentations, role-play, and practice sessions to standardize observation procedures and enhance interrater reliability.
- Observers independently documented care during labor, delivery, and simulated neonatal resuscitation, and **average scores were used for analysis**.

2. Sampling and Representation (Page 6, lines 12–28): The sample size justification is weak. Please provide power calculation (if available), or at least, and rationale for the selected sample size. Ethiopia is geographically and demographically diverse. Please explain whether certain regions were underrepresented, and if so, discuss the potential impact on the findings.

**Response:**

We thank the reviewer for this comment, and we provide clarification as follows:

**Sample size justification:**

- As it was already stated in the original manuscript, the sample size was determined using a **power analysis** to detect a **moderate effect size (d = 0.5)** with **80% power** and **α = 0.05**, ensuring sufficient power to detect medium-to-large differences between groups.
- This calculation resulted in **32 participants per group**.
- For the competency-based curriculum group, **32 of 37 recent DTU graduates** met the inclusion criteria; five were excluded due to unemployment, career change, or working outside the study area.
- For the comparison group, a 10% margin was added to account for anticipated non-response, resulting in a **final sample of 36 randomly selected participants** from the registrar lists of third-generation universities.

**Geographic representation:**

- Participants were employed across **all regions of Ethiopia**.
- Inclusion of both **public hospitals and health centers** serving **urban and rural populations** enhances contextual and demographic representativeness.
- Three of the nine third-generation universities were excluded: two had already implemented similar curricula, and one lacked sufficient data.

**Potential impact of underrepresentation:**

- While some universities were excluded due to curriculum overlap or unavailable data, the sample still captures a **diverse range of settings and regional representation**, minimizing selection bias.
- All participants had similar professional experience (<1 year) and had not received additional neonatal resuscitation or infection prevention training, reducing variability due to experience and strengthening the comparability of performance outcomes.

3. Linking Results to Policy and Practice (Page 11, lines 10–30): The manuscript claims that findings will inform national policy but does not describe the mechanisms for this. Suggestions: Explain how results could feed into curriculum reform, continuing professional development, or accreditation of midwifery programs, and provide specific recommendations for policymakers and professional associations.

**Response:** We appreciate the reviewer's comment.

- The Discussion now explicitly highlights how CBCGs’ improved performance demonstrates the potential of competency-based curricula to enhance clinical outcomes.
- The Conclusion frames a **testable and debatable argument**: competency-based curricula can improve clinical competence, but ongoing evaluation, targeted improvements, and context-specific adaptations are essential to fully realize their effectiveness.

These revisions clarify **how study results can inform curriculum reform, CPD initiatives, and accreditation processes**, providing actionable recommendations for policymakers, educational institutions, and professional associations. Therefore, these revisions explicitly link our findings to policy and practice implications as follows:

**Curriculum reform:**

- The superior performance of competency-based curriculum graduates (CBCGs) in neonatal resuscitation and infection prevention highlights the effectiveness of competency-based education.
- Policymakers and curriculum developers can use these findings to **scale up competency-based approaches**, emphasizing early clinical exposure, simulation-based training, and structured assessment to strengthen graduates’ critical clinical skills.
- The study supports the recommendation that curriculum reforms should incorporate **frequent simulation exercises, structured mentorship, and targeted feedback mechanisms** to address persistent skill gaps.

**Continuing Professional Development (CPD):**

- Observed deficiencies in critical steps (e.g., checking breathing/pulse, proper apron disinfection) suggest areas for **targeted CPD programs**.
- Health facilities and professional associations can design **refresher courses, mentorship programs, and competency-focused workshops** to reinforce essential skills among recent graduates.

**Accreditation and Quality Assurance:**

- Findings provide evidence to inform **national accreditation and program evaluation standards**, ensuring midwifery programs are assessed on measurable clinical competencies aligned with maternal and neonatal health priorities.
- Integration of competency-based performance metrics into accreditation processes can **ensure consistent quality of graduates nationwide**.

4. Statistical Transparency (Page 7–9): Tables and figures present data, but the analytical methods are not clearly reported. Clarify which tests (e.g., chi-square, t-tests, regression) were applied. Indicate levels of statistical significance (p-values) and confidence intervals.

**Response:** We appreciate the reviewer’s comment and would like to clarify that the manuscript already provides a detailed description of the statistical methods:

**Analytical approach:**

- Continuous outcomes (neonatal resuscitation and infection prevention scores) were compared using **independent samples t-tests**.
- **Effect sizes** (Cohen’s d) were calculated, and **Gardner–Altman plots** were used to visualize group differences.
- Assumptions of normality and homogeneity of variance were verified using **Shapiro–Wilk** and **Levene’s tests**, respectively.
- Multiple comparison corrections were applied using **Bonferroni, Holm–Bonferroni, and false discovery rate** methods.

**Statistical significance and confidence intervals:**

- All tests were two-tailed with **α = 0.05**.
- **P-values** and **95% confidence intervals** are reported in Tables 4 and in the Results section for both primary outcomes.

**Reliability:**

- Interrater reliability was assessed using a **two-way mixed-effects ICC** with excellent agreement (.84–.87).
- Internal consistency of the tools was evaluated using **Cronbach’s alpha** (.64–.66).

These details ensure full transparency, allowing readers to evaluate the robustness and precision of our findings.

5. Ethical Considerations (Page 4, lines 25–30): Ethical approval is mentioned, but informed consent procedures are not adequately explained. Authors should clarify how consent was obtained (oral/written), ensure anonymity/confidentiality, and confirm that participation was voluntary.

**Response:** We appreciate the reviewer's comment to this point. We have clarified the informed consent procedures in the Methods section:

1. **Ethical Approval:** The study was approved by the Ethical Review Committee of Debre Tabor University, Ethiopia (IRB No.: RP/251/23) and the Ethics Commission of the Medical Faculty at Ludwig-Maximilians-Universität, Munich, Germany (Project No.: 23-0757).
2. **Consent Procedures:**
   - Written informed consent was obtained from **facility directors**.
   - **Oral informed consent** was obtained from participating midwives and laboring women. Written consent was waived for midwives to reduce disruption during care, and for laboring women due to literacy barriers and to minimize burden during labor.
3. **Confidentiality and Voluntariness:**
   - Participation was fully voluntary, and midwives could decline observation without consequences.
   - Data were anonymized, facility and provider identifiers were omitted, and confidentiality was strictly maintained.

These procedures ensured ethical compliance while minimizing potential risks to participants.

**The *Ethical Considerations* section was revised as follows:**

*“The study protocol was approved by the Ethical Review Committee of Debre Tabor University, Ethiopia (Date: 13.07.2023 / No: RP/251/23), and the Ethics Commission of the Medical Faculty of Ludwig-Maximilians-Universität, Munich, Germany (Date: 18.10.2023 / Project Nr.: 23-0757). At each health facility, the supervisor presented an official letter from DTU to the facility director requesting cooperation. Written consent was obtained from facility directors, while oral consent was obtained from midwives and laboring women. Written consent was not collected from midwives due to their engagement in clinical care, although the potential benefits and burdens had been discussed beforehand. For laboring women, written consent was waived owing to literacy challenges and to minimize the burden during labor.*

*Data collectors were trained to approach participants respectfully, explain the study purpose, and obtain informed consent, including discussion of potential risks and benefits. Providers were assured that participation or refusal would not affect their employment, and women were assured that nonparticipation would not influence the care they received. Participant and facility confidentiality was strictly maintained: no names of facilities, service providers, or clients were recorded, and results were reported anonymously to prevent identification.*

*All informed consent procedures were reviewed and approved by the respective ethics committees.”*

**Minor Comments**

6. Formatting: Ensure tables/figures have complete captions. For example: Table 2 (Page 8) lacks sufficient explanation of abbreviations and context.

**Response:** We appreciate the reviewer's comment. We have updated all tables and figures, including Table 2, to provide full explanations of the abbreviations used. For Table 2, **“CCGs” clearly refers to Conventional Curriculum Graduates and “CBCGs” to Competency-Based Curriculum Graduates**. Other abbreviations, such as NR for Neonatal Resuscitation, are also defined in the table caption.

**Point 2: Context for the table:** We added context to Table 2 captions so that readers can understand **what the table presents, the type of measurement, and the setting of the assessment** without having to refer to the main text. The revision is indicated as follows.

*“Table 2. Performance of midwives in performing steps of neonatal resuscitation during simulated resuscitation by type of preservice curriculum in Ethiopia (N = 68).”*

7. References: Several references are outdated (pre-2010). Please incorporate recent literature on midwifery education and competencies, particularly from Ethiopia and sub-Saharan Africa (e.g., WHO 2021 State of the World’s Midwifery).

**Response:**
We thank the reviewer for highlighting the need to update the references. We carefully reviewed our reference list, and outdated references were either updated with more recent studies or retained only where no newer evidence exists.

8. Grammar/Style: Numerous sentences could be shortened for clarity. Example: Page 3, line 22 – “The framework is very comprehensive and globally used by many institutions” → “The framework is comprehensive and widely adopted globally.”

**Response:**
We thank the reviewer for the suggestion to improve clarity and readability. ***The specific example cited does not appear in our manuscript***. We have carefully reviewed the manuscript and revised sentences throughout to shorten overly long statements, remove redundancy, and improve overall readability.

9. Terminology Consistency: The term “competency gap” is sometimes used interchangeably with “knowledge gap.” Clarify the distinction.

**Response:** We appreciate the reviewer's suggestion. We carefully reviewed the full original manuscript and searched for “competency gap,**” and we confirmed that it** does **not appear anywhere** in the manuscript.

10. Ethical and Publication Concerns: No evidence of dual publication or plagiarism was identified.

**Response:** We thank the reviewer for confirming that there are no ethical or publication concerns. We also confirm that this manuscript is original, has not been published elsewhere, and is not under consideration by any other journal.

11. Ethical approval is reported, but more detail on informed consent is required for compliance with international research ethics standards.

**Response:**
We thank the reviewer for highlighting the need for more detail. We have revised the Methods section (***Section 2.7, Ethical Considerations***) to provide a clear description of the informed consent procedures.

**Recommendation:**
The manuscript has strong potential to contribute to global knowledge on midwifery workforce development, particularly in low-resource settings. However, substantial revisions are necessary to clarify and strengthen methodology, justify sampling and representation, provide transparent statistical reporting, strengthen the link between findings and actionable policy implications, and revise language for clarity and flow.

If these revisions are addressed, the manuscript could make a meaningful and publishable contribution to maternal and newborn health systems research.

**Response:** We thank the reviewer for acknowledging the potential contribution of our study. We believe that we have carefully addressed all comments and made adequate revisions as follows:

- **Methodology Clarification:** We have provided additional details on study design, data collection procedures, and outcome measurement tools, including observer training, simulation protocols, and inter-rater reliability procedures.
- **Sampling and Representation:** We clarified the inclusion/exclusion criteria, sample size calculation, and rationale for selecting comparison universities to ensure representativeness of third-generation Ethiopian midwifery graduates.
- **Statistical Reporting:** We have revised the Results section to include clear and transparent reporting of t-tests, effect sizes (Cohen’s d), confidence intervals, and multiple comparison corrections, along with visualizations via Gardner–Altman plots.
- **Policy Implications:** The Discussion and Conclusion sections now explicitly link our findings to actionable recommendations for midwifery education policy and workforce development, emphasizing simulation-based training, structured mentorship, and curriculum improvement.
- **Language, Clarity, and Flow:** The manuscript has been carefully revised for readability, conciseness, and consistency in terminology (e.g., distinguishing “competency gap” from “knowledge gap”), ensuring smooth narrative flow throughout the text.

We believe these revisions substantially strengthen the manuscript and address the reviewer’s concerns, enhancing its contribution to maternal and newborn health systems research in low-resource settings.
