# Supplementary material for: Evaluating the effectiveness of preservice midwifery curricula in Ethiopia: A comparison of neonatal resuscitation and infection prevention practice of midwifery graduates trained in competency-based versus conventional curricula
Source: PLoS One. 2026 Feb 10;21(2):e0338395. doi: 10.1371/journal.pone.0338395 (PMC12890084; doi:10.1371/journal.pone.0338395)
Supplement: Attachment — Submitted filename: Response to Reviewers III _ III.docx [file pone.0338395.s006.docx]

**To:** The Editorial Office

**PLOS ONE**

**Subject:** Submission of Revised Manuscript “***Evaluating the effectiveness of preservice midwifery curricula in Ethiopia: A comparison of neonatal resuscitation and infection prevention practice of midwifery graduates trained in competency-based versus conventional curricula (PONE-D-25-43421*)**”

Dear Editors,

On behalf of all authors, I am pleased to submit the revised version of our manuscript entitled *“Evaluating the effectiveness of preservice midwifery curricula in Ethiopia: A comparison of neonatal resuscitation and infection prevention practice of midwifery graduates trained in competency-based versus conventional curricula”* for consideration in **PLOS ONE**.

We thank the reviewer for the thoughtful feedback and constructive suggestions. In this revision, we have addressed all comments.

We believe that these revisions substantially improve the clarity, rigor, and impact of the manuscript. We are confident that this study provides important evidence on the effectiveness of competency-based midwifery curricula in Ethiopia and contributes to the global literature on the development of the maternal and newborn health workforce.

This manuscript is original, has not been published elsewhere, and is not under consideration by any other journal. All authors have reviewed and approved the final version.

I kindly wish to bring to your attention that I applied for a publication fee scholarship whose deadline was November 15. Although my application was submitted on time, the scholarship office has informed me that they require confirmation of the manuscript’s decision status in order to reserve the funds. Without this confirmation, the financial support may no longer be available.

In light of this, I would be sincerely grateful if the decision process could be finalized as soon as feasible, or if an interim confirmation could be provided. This would greatly assist me in securing the necessary funding for publication.

We greatly appreciate your time and consideration and look forward to your favorable response.

Sincerely,
on behalf of all co-authors,

**Awoke Giletew Wondie, MHPE, MPH**

PhD Candidate in Medical Research – International Health

Ludwig-Maximilians-Universität München

**Journal Requirements:**

1. If the reviewer comments include a recommendation to cite specific previously published works, please review and evaluate these publications to determine whether they are relevant and should be cited. There is no requirement to cite these works unless the editor has indicated otherwise. 
Not recommended.

2. Please review your reference list to ensure that it is complete and correct. If you have cited papers that have been retracted, please include the rationale for doing so in the manuscript text, or remove these references and replace them with relevant current references. Any changes to the reference list should be mentioned in the rebuttal letter that accompanies your revised manuscript. If you need to cite a retracted article, indicate the article’s retracted status in the References list and also include a citation and full reference for the retraction notice.

**Response:** We have carefully revised the reference; no change has been made.

**Additional Editor Comments:**

Please find a few additional minor comments from the reviewer. Kindly review and respond to them.

**Comments to the Author**
1. If the authors have adequately addressed your comments raised in a previous round of review and you feel that this manuscript is now acceptable for publication, you may indicate that here to bypass the “Comments to the Author” section, enter your conflict of interest statement in the “Confidential to Editor” section, and submit your "Accept" recommendation.

Reviewer #1: All comments have been addressed

Reviewer #3: (No Response)

2. Is the manuscript technically sound, and do the data support the conclusions?

The manuscript must describe a technically sound piece of scientific research with data that supports the conclusions. Experiments must have been conducted rigorously, with appropriate controls, replication, and sample sizes. The conclusions must be drawn appropriately based on the data presented.

Reviewer #1: Yes

Reviewer #3: Yes

3. Has the statistical analysis been performed appropriately and rigorously?

Reviewer #1: Yes

Reviewer #3: Yes

4. Have the authors made all data underlying the findings in their manuscript fully available?
The [PLOS Data policy](http://track.editorialmanager.com/CL0/http:%2F%2Fwww.plosone.org%2Fstatic%2Fpolicies.action%23sharing/1/010f019a99c0a2d4-6d896646-3577-4939-84be-644f0b4b2f45-000000/EVGyJvKucvYxVaC16swiSx97SoWu85A5Dg2UYoqgI6w=237) requires authors to make all data underlying the findings described in their manuscript fully available without restriction, with rare exception (please refer to the Data Availability Statement in the manuscript PDF file). The data should be provided as part of the manuscript or its supporting information, or deposited to a public repository. For example, in addition to summary statistics, the data points behind means, medians and variance measures should be available. If there are restrictions on publicly sharing data—e.g. participant privacy or use of data from a third party—those must be specified.

Reviewer #1: Yes

Reviewer #3: Yes

5. Is the manuscript presented in an intelligible fashion and written in standard English?

PLOS ONE does not copyedit accepted manuscripts, so the language in submitted articles must be clear, correct, and unambiguous. Any typographical or grammatical errors should be corrected at revision, so please note any specific errors here.

Reviewer #1: Yes

Reviewer #3: Yes

6. Review Comments to the Author

Please use the space provided to explain your answers to the questions above. You may also include additional comments for the author, including concerns about dual publication, research ethics, or publication ethics. (Please upload your review as an attachment if it exceeds 20,000 characters)

**Reviewer #1**: Based on a thorough review of the revisions for manuscript, the authors have comprehensively and satisfactorily addressed all points/feedbacks. The revisions demonstrate a high degree of scholarly diligence and have resulted in a fundamental improvement of the manuscript.

**Key enhancements include:**
Conceptual Reframing: The study has been successfully elevated from a descriptive analysis to a rigorous, hypothesis-driven piece of scientific research with a clear and debatable thesis.
Methodological Transparency: The manuscript now features increased detail on the study's methodology, enhancing its clarity and replicability.

Contextual Depth: The discussion has been significantly strengthened with a more nuanced analysis of the findings, including persistent skill gaps and the influence of the post-graduation work environment.

The collective impact of these changes is a manuscript with a sharpened argumentative focus that represents a more significant and durable contribution to the field of midwifery education. The work now meets a high standard of scientific communication.

**Response to Reviewer 3**

Reviewer #3: This is an important study for LMIC's in light of reports of poor maternal-child health outcomes, and SDG 3, especially the role of midwives. Thank you for your initiative. I am recommending that the manuscript be accepted after these minor but important corrections have been addressed.
I just have a few suggestions related to ethics, and grammar, respectively: Researchers had ample opportunity to request a written consent from midwives during the planning phase unless this was meant to be a surprise assignment. Midwives could have been asked to put in writing their willingness to participate in the study rather than being told by their administrators, who signed a consent allowing researchers to conduct the study. My understanding is that it was the midwives who were being observed so their written consent could have been obtained in advance for that study period. For the women in labor, illiteracy is not a sufficient reason for there being no written consent. They could have been asked to place a check mark ("my mark") on the signature line after the "informed consent" had been read to them. Understandably, seeking care at a facility could be interpreted as implied consent however, research is not routine care, and all attempts ought to be made for objectivity and accountability.

**Response:**

Thank you very much for this thoughtful and important comment regarding ethical procedures. We appreciate the reviewer’s emphasis on strengthening consent processes, particularly in research involving frontline health workers and women in labor.

1. **Written consent from midwives**

We agree that written consent is generally preferred. In our context, however, the ethics committees approved oral consent for midwives due to the nature of their clinical duties. Midwives were providing continuous intrapartum care, and requiring written signatures during or immediately prior to clinical shifts was considered potentially disruptive to patient care. Instead, midwives were approached individually outside the observation moment, informed fully about the study, and provided oral consent voluntarily. Importantly, midwives were explicitly informed that participation was not mandatory, and administrators were not involved in the consent process to avoid coercion. We acknowledge the reviewer’s point and have added clarification in the manuscript.

2. **Written consent for laboring women**

We appreciate the reviewer’s concerns regarding accountability and objectivity. As clarified in the revised manuscript, the ethics committees waived written consent for laboring women because obtaining signatures while in active labor was deemed inappropriate and posed potential distress. Women were instead provided verbal explanations during periods of comfort, and consent was recorded orally with approval from both ethics committees. We agree with the suggestion regarding the possible use of non-signature marks; however, this was evaluated during planning and deemed potentially burdensome during active labor. We have now clarified this rationale more explicitly in the revised text.

3. **Revision added**

We have revised the Ethical Considerations section to better explain the decision-making process, the ethics committee approval for oral consent, and the safeguards taken to ensure voluntary participation and non-coercion.

**Revised Section 2.7 Ethical Considerations**

*The study protocol was approved by the Ethical Review Committee of Debre Tabor University, Ethiopia (Date: 13.07.2023 / No: RP/251/23), and the Ethics Commission of the Medical Faculty of Ludwig-Maximilians-Universität, Munich, Germany (Date: 18.10.2023 / Project Nr.: 23-0757). At each health facility, the supervisor presented an official letter from DTU to the facility director requesting cooperation. For midwives, both ethics committees approved the use of oral informed consent. Although written consent is generally preferred, requiring signatures during clinical shifts was determined to risk disrupting urgent intrapartum care. To minimize coercion and ensure voluntariness, midwives were approached individually, outside the immediate care setting, and informed about the study's purpose, risks, and benefits. They were also provided the opportunity to decline participation without any involvement from facility administrators. Their oral consent was documented by trained data collectors as approved in the ethical protocol.*

*For laboring women, the ethics committees granted a waiver of written consent. Many women were in active labor or experiencing discomfort, and requesting written signatures, including check marks or symbolic signatures, was considered to impose an additional burden during a vulnerable moment. Instead, data collectors read the consent information aloud in the woman’s preferred language, answered questions, and obtained verbal consent, which was recorded in the data collection system. Women were assured that refusal would not affect the care they received. Across all participants, confidentiality was strictly maintained: no identifying information about midwives, women, or facilities was collected, and all results were reported anonymously. All consent procedures were conducted in accordance with approved protocols, with an emphasis on minimizing burden, respecting autonomy, and safeguarding participants’ rights.*

*All informed consent procedures were reviewed and approved by the respective ethics committees.*

**Grammar:**

1) **Abstract line 25... add "can" reduce ...**

**Response:** Thank you for this helpful suggestion. We agree that adding “can” improves accuracy and avoids overstating the causal relationship. The sentence has been revised accordingly in the abstract.

**Original:**
“Infection control and neonatal resuscitation are essential midwifery practices that reduce maternal and neonatal mortality.”

**Revised:**
“Infection control and neonatal resuscitation are essential midwifery practices that **can** reduce maternal and neonatal mortality.”

2) **line 57-58 sequence of actions for preventing infection: it reads better if "early detection of maternal infection" e.g. in the prenatal period, comes before "...using sterile techniques..."
   Response:** Thank you for this valuable suggestion. We agree that reordering the sequence improves the logical flow by placing early detection in the prenatal or early intrapartum period before infection prevention procedures during labor. We have revised the sentence accordingly.

Original: “Midwives play a central role in preventing infections through sterile techniques during labor and delivery, and early detection of maternal infections.”

Revised: “Midwives play a central role in preventing infections through early detection of maternal infections, and by using sterile techniques during labor and delivery.”

3) **line 101 "data [were] collected" as was done later in the report.**

**Response:** Thank you for noting this inconsistency in verb agreement. We have corrected the phrase to use the plural form “were collected,” ensuring grammatical consistency throughout the manuscript.

**Original:**
“Data was collected from August 26 to October 12, 2024.”

**Revised:**
“Data **were** collected from August 26 to October 12, 2024.”

4) **line 111 statistics- include confidence interval 95% after ..." 80% power" as was mentioned in the data analysis section of the report.**

**Response:** Thank you for this helpful suggestion. We agree that including the reference to the 95% confidence interval enhances clarity and aligns the sample size description with the reporting style used later in the analysis section. We have revised the sentence accordingly.

Original:
“The number of participants was calculated based on a power analysis to determine a moderate effect size (d = 0.5) with 80% power and α = .05, ensuring sufficient power to detect a medium-to-large effect size between groups…”

Revised:
“The number of participants was calculated based on a power analysis to determine a moderate effect size (d = 0.5) with 80% power, a 95% confidence level, and α = .05, ensuring sufficient power to detect a medium-to-large effect size between groups…”

5) **line 136 remove /" ... not performed/correctly"**

**Response:** Thank you for this observation. We agree that the phrase “not performed/correctly” is unnecessary and may cause ambiguity. We have removed it to improve clarity and readability.

**Original:**
“Each task was scored as ‘performed correctly’ or ‘not performed/correctly’ to calculate a percentage performance score.”

**Revised:**
“Each task was scored as ‘performed correctly’ or ‘not performed’ to calculate a percentage performance score.”

6) **line 151 suggest writing IBM Statistical Package for the Social Services (SPSS) this being the first time it comes up in the report.**

**Response:** Thank you for this helpful suggestion. We agree that the full name of the software should be provided at first mention for clarity and formality. We have revised the sentence accordingly.

**Original:**
“Data were analysed using IBM SPSS Statistics version 27.”

**Revised:**
“Data were analysed using **IBM Statistical Package for the Social Sciences (SPSS) Statistics**, version 27.”

7) **Results- consistency in usage of verb/tense e.g. "Dry", "Assess", "Check"**

**Response:** Thank you for this important observation. We agree that the Results section should maintain consistent verb tense and form, particularly within tables and descriptions of observed actions. To address this, we revised action verbs in Tables 2 and 3 and the surrounding narrative so that all procedural steps begin with consistent base-form verbs (e.g., “Dries,” “Assesses,” “Checks”). These adjustments improve clarity and ensure grammatical consistency throughout the Results section.

Original read:

- “Dry the baby…”
- “Assess for spontaneous breathing”
- “Check pulse/heartrate”

Revision:

- “Dries the baby and wraps in a dry, warm cloth”
- “Assesses for spontaneous breathing”
- “Checks pulse/heart rate”
